# Species-specific marker development for accurate identification of three red algae (*Grateloupia asiatica*, *Pachymeniopsis lanceolata* and *Polyopes affinis*) based on complete organelle genomes

**DOI:** 10.1007/s42995-025-00327-4

**Published:** 2025-10-28

**Authors:** Yong Jin Lee, Yeon Mi Kim, Cheol Min Kim, Sung-Je Choi, Cheol Seong Jang

**Affiliations:** 1 Agriculture and Life Sciences Research Institute, Kangwon National University, Chuncheon, 24341 Korea; 2 Plant Genomics Laboratory, Interdisciplinary Program in Smart Agriculture, Kangwon National University, Chuncheon, 24341 Korea; 3 Department of Aquaculture and Fisheries Convergence, Korea National College of Agriculture and Fisheries, Jeonju, 54874 Korea

**Keywords:** Halymeniaceae, Chloroplast genome, Mitochondrial genome

## Abstract

Red alga species in the phylum Rhodophyta are ecologically and economically important, which are widely used as food and medicinal products because they are rich in bioactive compounds. Unfortunately, researchers often meet challenges in identifying red algal species and understanding the evolutionary relationship among them. Shooting away these limitations necessitates detailed genomic studies, including those of cellular organelles. In this study, we sequenced circular organelle genomes of three red macroalgal species, *Grateloupia asiatica*, *Pachymeniopsis lanceolata* and *Polyopes affinis*, yielding two complete chloroplast genomes of *P. lanceolata* and *P. affinis* and one mitochondrial genome of *P. affinis*. The average chloroplast and mitochondrial genome sizes were 192,724 bp and 29,699 bp, respectively, which encoded 202 and 25 proteins, respectively, on average. The short- and long-repeat sequences, gene rearrangements, the nucleotide diversity, and phylogenetic relationship among these three species were analyzed. Memo: revise conjunctions and prepositions to form a clearer list (A, B, C, and D). The 194 chloroplast and 23 mitochondrial protein-coding genes shared by the species belonging to Florideophyceae and Bangiophyceae were used to reconstruct a maximum-likelihood phylogenetic tree. In addition, we developed 15 species-specific PCR markers, five for each species, using single-nucleotide polymorphism information. Our results should aid in identifying these species and deciphering the evolutionary relationship among species in the phylum Rhodophyta.

**Supplementary Information:**

The online version contains supplementary material available at 10.1007/s42995-025-00327-4.

## Introduction

Florideophyceae, a major class of red algae in phylum Rhodophyta, contributes about 95% of the total diversity of the phylum Rhodophyta, which encompasses > 7500 documented species in AlgaeBase (https://www.algaebase.org/) (Guiry 2024). These red algal species play pivotal roles in diverse marine ecosystems and have been extensively used in food, pharmaceutical and biotechnological industries; they are rich in high-value bioactive compounds (Cui et al. 2019; Kandale et al. 2011; Lee et al. 2023; Villanueva et al. 2014). The global demand for red alga-derived products is increasing constantly, and accordingly the sustainable exploitation of these red algal species requires accurate species identification.

Researchers have met the challenges in identifying these red algal species. The traditional methods often fail to distinguish them due to intra- and inter-specific variations, simple and convergent morphologies and phenotypic plasticity with environments (Kim et al. 2023; Saunders 2005; Verlaque et al. 2005). Occasional modifications in the scientific names of red algal species like *Porphyra dentata* (now *Neoporphyra dentata*) and *Grateloupia lanceolata* (now *Pachymeniopsis lanceolata*) have also complicated the identification of red algal species, which is even worsened by the frequent application of outdated names (Lee et al. 2023; Li et al. 2019; Yang et al. 2022). In addition, morphological characteristics alone are often insufficient for the accurate identification of Rhodophyta species; they may lead to unintended mixing or misuse of different species during manufacturing processes (Yang and Kim 2015). To address these challenges, the species boundaries and genetic relationships among Rhodophyta species should be untangled using molecular tools and genomic resources.

The genomes of chloroplasts and mitochondria promise to be the resources for winning these challenges. These genomes are relatively small and high in copy numbers, making them extremely applicable for developing species-specific molecular markers (Greiner et al. 2015; Liu et al. 2024; Rawal et al. 2020; Wang et al. 2021). However, the organelle genomic data for Florideophyceae remain limited, hindering our endeavors to develop reliable markers for species identification.

Recent advancements in high-throughput sequencing technologies have made the analysis of organellar genomes convenient. These technologies allow researchers to characterize the genomes in their gene content, structural variation, repeat element identification among others, supporting the development of species-specific PCR primers.

In this study, we enriched the genomic resources of economically important red algae by newly sequencing the organelle genomes of three species, *Grateloupia asiatica*, *Pachymeniopsis lanceolata* and *Polyopes affinis*. These species are used in South Korea as edible seaweeds. They are known also for their health benefits such as anti-inflammation and atopic dermatitis treatment (Ha et al 2022; Hwang and Park 2020; Khan et al 2008). These species were selected as the studying materials due to their economic significance and health application and less described genomic characteristics. The primary goal of this study was to develop reliable species-specific markers for accurate species identification. We sequenced and assembled the organellar genomes of these three species, and then conducted comparative analyses and deciphered phylogenetic relationships among species in Florideophyceae. We analyzed their mitochondrial (mt) genomes to reinforce the accuracy of their classification. We subsequently developed species-specific markers based on the SNPs found among the chloroplast (cp) genomes. We generated the first complete cp genomes for *P. lanceolata* and *P. affinis* and the first mt genome for *P. affinis*. Our results bridged the gaps between the genomic data and the species identification, and provided the foundation to the species identification. Furthermore, our findings enabled further explorations of the organelle genomes of other red algal species.

## Materials and methods

### Plant materials and DNA isolation

Algal samples of *G. asiatica*, *P. lanceolata* and *P. affinis* were collected from the coastal area of Jeju Island, Republic of Korea. The collection of algal samples was permitted by the Ministry of Ocean and Fisheries, Republic of Korea. This study was conducted in compliance with local, national, Kangwon National University (Chuncheon, Republic of Korea), and Ministry of Oceans and Fisheries (Sejong, Republic of Korea) regulations. The collected samples were identified by professional experts on Korean algae. The total DNA was extracted using the Exgene Plant SV Kit (GeneAll, Seoul, Republic of Korea) following the manufacturer’s directions.

### Complete genome construction and annotation

Genomic library construction and DNA sequencing were performed using an Illumina NovaSeq 6000 Platform yielding 151 bp paired-end reads by Macrogen Inc. (Seoul, Republic of Korea). Circular organelle genomes were constructed by comparing and integrating two assemblers, NOVOPlasty and GetOrganelle (Dierckxsens et al. 2017; Jin et al. 2020c) and with one mapping tool, Geneious Prime software. The adapter sequences were trimmed using fastp (Chen et al. 2018) for NOVOPlasty following the developer’s recommendation, and quality filtering was conducted for GetOrganelle and Geneious Prime. The reference sequences were provided for Geneious Prime (mandatory) and NOVOPlasty (optional). For the construction of the organelle genomes of newly completed *G. asiatica* and *P. lanceolata* in this study, we used the *G. asiatica* cp genome (AP018129.2) and mt genome (OR221180.1) as the references. For *P. affinis*, the *Halymenia maculata* chloroplast genome (NC_046751.1) and the *P. affinis* mitochondrial genome (NC_063816.1) were used as references. The *rbcL* and *cox3* genes were used as the seeds in assembling cp and mt genomes, respectively.

Genome annotation was performed by comparing the annotations from two tools, GeSeq (Tillich et al. 2017), a web-based organelle genome annotation tool, and Geneious Prime. The tRNAscan-SE (Chan and Lowe 2019) was used to predict tRNA genes, and OGDRAW (Greiner et al. 2019) was used to draw organelle genome maps. GeSeq, including tRNAscan-SE and OGDRAW, was performed on the website https://chlorobox.mpimp-golm.mpg.de/index.html. The names and symbols of the genes were manually standardized by comparing the organelle genomes across the species used in this study.

### Repeat sequence analysis

Simple sequence repeats (SSRs) were identified using MISA (Beier et al. 2017) with the parameters set 10 > for mono-, > 5 for di- and tri-, and > 3 for tetra-, penta-, and hexa-nucleotide. Dispersed long repeats of four types (forward, palindromic, reverse and complementary) were calculated using REPuter (Kurtz et al. 2001) with the parameters of hamming distance 3, maximum computed repeats 50, and minimal repeat size 30 bp.

### Comparative analysis

To detect the rearrangements, inversions and synteny of genes among Florideophyceae species, the progressiveMauve (Darling et al. 2004) implemented in Geneious was used with the newly assembled *G. asiatica* organelle genome (OR635816.1 for cp genome and OR635819.1 for mt genome) as the references. The similarities of the newly assembled organelle genomes of the three species were plotted using the mVISTA web-based program with Shuffle-LAGAN mode using the *G. asiatica* organelle genomes (AP018129.2 for cp genome and OR221180.1 for mt genome) downloaded from NCBI as the references. To estimate the nucleotide diversity value (Pi), the organelle genomes of the three species were aligned using MAFFT v7.520 (Katoh and Standley 2013), and then a sliding window analysis was performed using DnaSP v6.12.03 (Rozas et al. 2017). The window length was set to 300 bp for cp genomes and 200 bp for mt genomes, and the step size was set to 200 bp for cp genomes and 20 bp for mt genomes. Relative synonymous codon usage (RSCU) was analyzed for all protein-coding genes (PCGs) using CodonW program (http://codonw.sourceforge.net/) and visualized with Phylosuite (Zhang et al. 2020).

To investigate the selective pressure on the PCGs of organelle genomes, the amino acid sequences of the PCGs were aligned using MUSCLE v5.1 (Edgar 2022), and the aligned sequences were converted to nucleotide sequences using PAL2NAL (Suyama et al. 2006) with the nogap option. The alignment files were converted to pairwise aligned axt format by the script AXTConvertor implemented in KaKs_Calculator 2.0 (Wang et al. 2010), and the files were used to calculate non-synonymous (Ka), synonymous (Ks) substitutions and Ka/Ks value with Yang–Nielsen (YN) algorithm calculation method.

### Phylogenetic analysis

The organelle genome data of 15 species in class Florideophyceae, 12 species in Bangiophyceae, a sister taxon of Florideophyceae, and one in Compsopogonophyceae, *Compsopogon caeruleus* as the outgroup were retrieved from NCBI GenBank to reconstruct the phylogenetic trees (Supplementary Table S1). The 194 cp and 23 mt PCGs (Supplementary Table S2) shared by the species were aligned using MAFFT, trimmed using trimAl (Capella-Gutiérrez et al. 2009), and concatenated using SequenceMatrix (Vaidya et al. 2011). Maximum-likelihood (ML) phylogenetic trees were constructed using IQ-TREE 2 (Minh et al. 2020) with 1000 bootstrap replicates, 1000 SH-aLRT replicates, and an approximate Bayes test. The best-fit models were chosen by ModelFinder implemented in IQ-TREE 2 using the option “-m TESTNEW”.

### Development of species-specific markers

The single-nucleotide polymorphisms (SNPs) of the aligned PCG sequences were detected, and the Beacon Designer (PRIMER Biosoft, Palo Alto, CA, USA) was used to design species-specific primer pairs based on polymorphic sites. qRT-PCR was performed on a Quantstudio 3 Real-Time PCR System (Applied Biosystems, Foster City, CA, USA) with AccuPower® 2X GreenStar qPCR Master Mix (Bioneer, Daejeon, Korea).

## Results

### Characteristics of the organelle genomes of *Grateloupia asiatica*, Pachy*meniopsis lanceolata* and *Polyopes affinis*

**Fig. 1:**
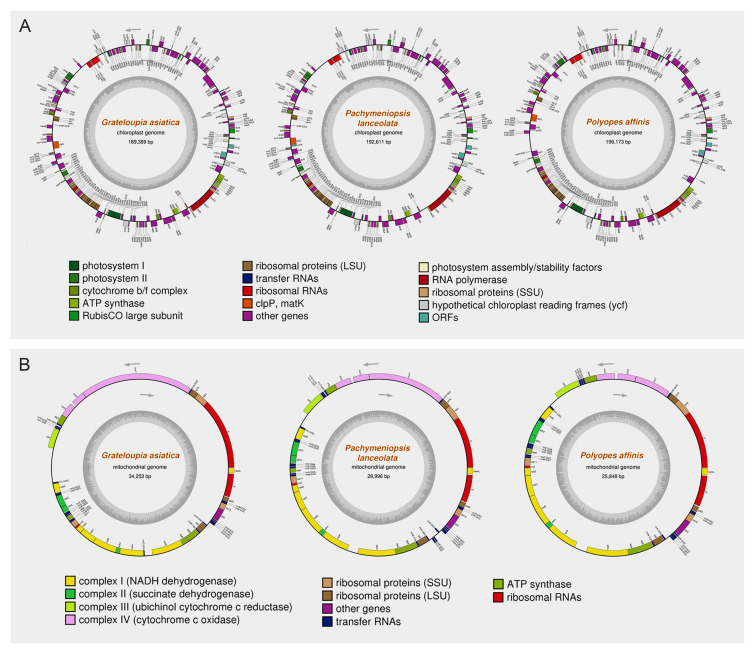
The complete circular genome maps of chloroplast **A** and mitochondria **B** of three species (*Grateloupia asiatica*, *Pachymeniopsis lanceolata*, and *Polyopes affinis*, from left to right). Genes inside and outside of the maps are transcribed in a clockwise and counterclockwise direction

For the three newly sequenced species, Illumina PE sequencing generated 100,694,720 (*G. asiatica*), 123,689,536 (*P. lanceolata*), and 124,205,314 (*P. affinis*) raw reads. Complete circular organelle genomes of the three species were constructed by comparing and combining the results of two assemblers, GetOrganelle and NOVOPlasty, and Geneious mapping (Fig. 1). These two assemblers sometimes include ambiguous nucleotides in their results and fail to generate a complete genome sequence. Additionally, in the case of mapping, a deletion in a genome relative to the reference sequence can result in addition of sequence to the map of the corresponding region. In our previous studies, we observed a deletion in the rRNA region (one *rrs* and one *rrl*) of *N. seriata* compared to that of other two species, *N. yezoensis* and *N. dentata*. However, unlike the results of the two assemblers, the mapping results placed virtual sequences in the deleted region (Lee et al. 2023). We thus compared the results of these three methods to derive the complete sequence. The cp and mt genomes of *P. lanceolata* and the cp genome of *P. affinis* (OR635818.1, OR635821.1, and OR635817.1, respectively) were documented for the first time here.

According to the yield of NOVOPlasty assembler, the coverage varied among the organelle genomes of three species (*G. asiatica* cp, 1842X; *P. lanceolata* cp, 3536X; *P. affinis* cp, 8416X; *G. asiatica* mt, 2148X; *P. lanceolata* mt, 2447X; *P. affinis* mt, 10,276X). The cp genome size was 189,389 bp (*G. asiatica*), 192,611 bp (*P. lanceolata*) and 196,173 bp (*P. affinis*), respectively. The average GC content was 29.93%. Across three species, there were 206 PCGs, 29 tRNA genes and three rRNA genes (Table 1). Although tRNA genes and rRNA genes were located identically across three species, some missing PCGs were found between species. Among a total of 206 PCGs, two genes in *G. asiatica* (*orf16* and *orf18*), four genes in *P. lanceolata* (*orf13*, *orf16*, *orf24*, and *orf48*) and seven genes in *P. affinis* (*grx*, *orf13*, *orf18*, *orf25*, *orf48*, *ycf21*, and *ycf56*) were missing.

**Table 1:** Basic characteristics of complete organelle genomes of the three species

Organelle	Characteristic	*Grateloupia asiatica*	*Pachymeniopsis lanceolata*	*Polyopes affinis*
Chloroplast	Genome size (bp)	189,389	192,611	196,173
	GC content (%)	30.3	30.2	29.3
	Protein coding genes no.	204	202	199
	tRNA genes no.	30	29	29
	rRNA genes no.	3	3	3
Mitochondria	Genome size (bp)	26,543	32,498	35,863
	GC content (%)	30	31.9	32.4
	Protein coding genes no.	24	25	26
	tRNA genes no.	24	24	24
	rRNA genes no.	2	2	2

The average length of mt genomes was 29,699 bp (34,253 bp for *G. asiatica*; 28,996 bp for *P. lanceolata*; 25,848 bp for *P. affinis*) with an average GC content of 31.87%. All mitochondrial genomes contained 25 unique PCGs and three rRNA genes. A total of 22 tRNA genes were found among mt genomes, while *trnR-UCU*, *trnS-GCU* and *trnY-GUA* were found only in *P. lanceolata* mt genome. All mt genomes had two copies of *trnM-CAU*, and *G. asiatica* had two copies of *trnC-GCA*.

All organellar genes are listed and divided into several categories according to their functions (Supplementary Table S3). The cp genes were divided into seven categories, genetic system, photosystems, ATP synthesis, metabolism, transport, RNA genes and unknown, and further divided into 26 groups. The mt genes were divided into four categories, oxidative phosphorylation, genetic system, RNA genes and transport, and 10 groups.

### SSRs and repeats analysis

**Fig. 2:**
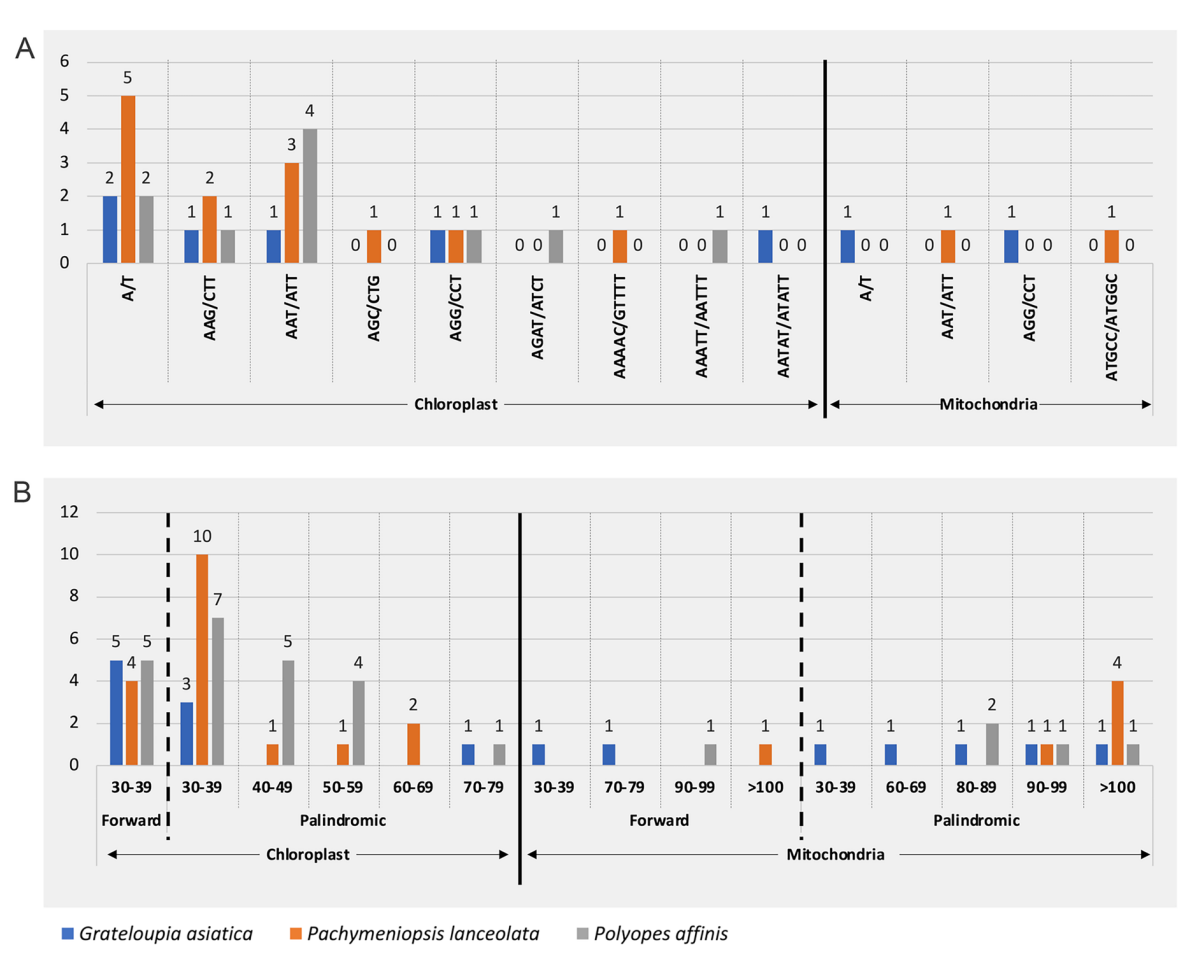
Comparison of the repeat sequences of the organelle genomes of three species. **A**: units and number of simple sequence repeats (SSRs). X-axis: repeat units; Y-axis: frequency. **B**: Long and interspersed repeats. X-axis: repeat length; Y-axis: copy number

Among cp genomes, six (*G. asiatica*), 13 (*P. lanceolata*) and 10 (*P. affinis*) SSRs with a total of nine repeat units were identified. The most abundant repeat sequence was A/T, and G/C repeats were not identified. SSRs of the di-nucleotide type were not found among the three species. Tri-nucleotide repeat types, AAG/CTT, AAT/ATT, AGC/CTG and AGG/CCT, were the most abundant, and a total of 16 repeats were found. One tetra- and three penta-nucleotide repeat units were found only once in cp genomes, AGAT/ATCT and AAATT/AATTT of *P. affinis*, AAAAC/GTTTT of *P. lanceolata*, and AATAT/ATATT of *G. asiatica*. The repeat units in the mt genomes were found only once (A/T and AGG/CCT in *G. asiatica*, and AAT/ATT and ATGCC/ATGGC in *P. lanceolata*), and no repeats were found in the mt genome of *P. affinis* (Fig. 2A).

Although REPuter was set up to detect four types (forward, palindromic, reverse, and complementary) of repeats, only two (forward and palindromic) were found in the organellar genomes of three species (Fig. 2B). Repeats in the range of 30 to 39 bp were identified to be the most abundant repeats in both forward and palindromic repeat types in cp genomes. For most repeats found in mt genomes, one repeat per length ranges was identified, except for 80–89 bp in *P. affinis* and > 100 bp in *P. lanceolata*.

### Comparative analysis of organelle genomes

**Fig. 3:**
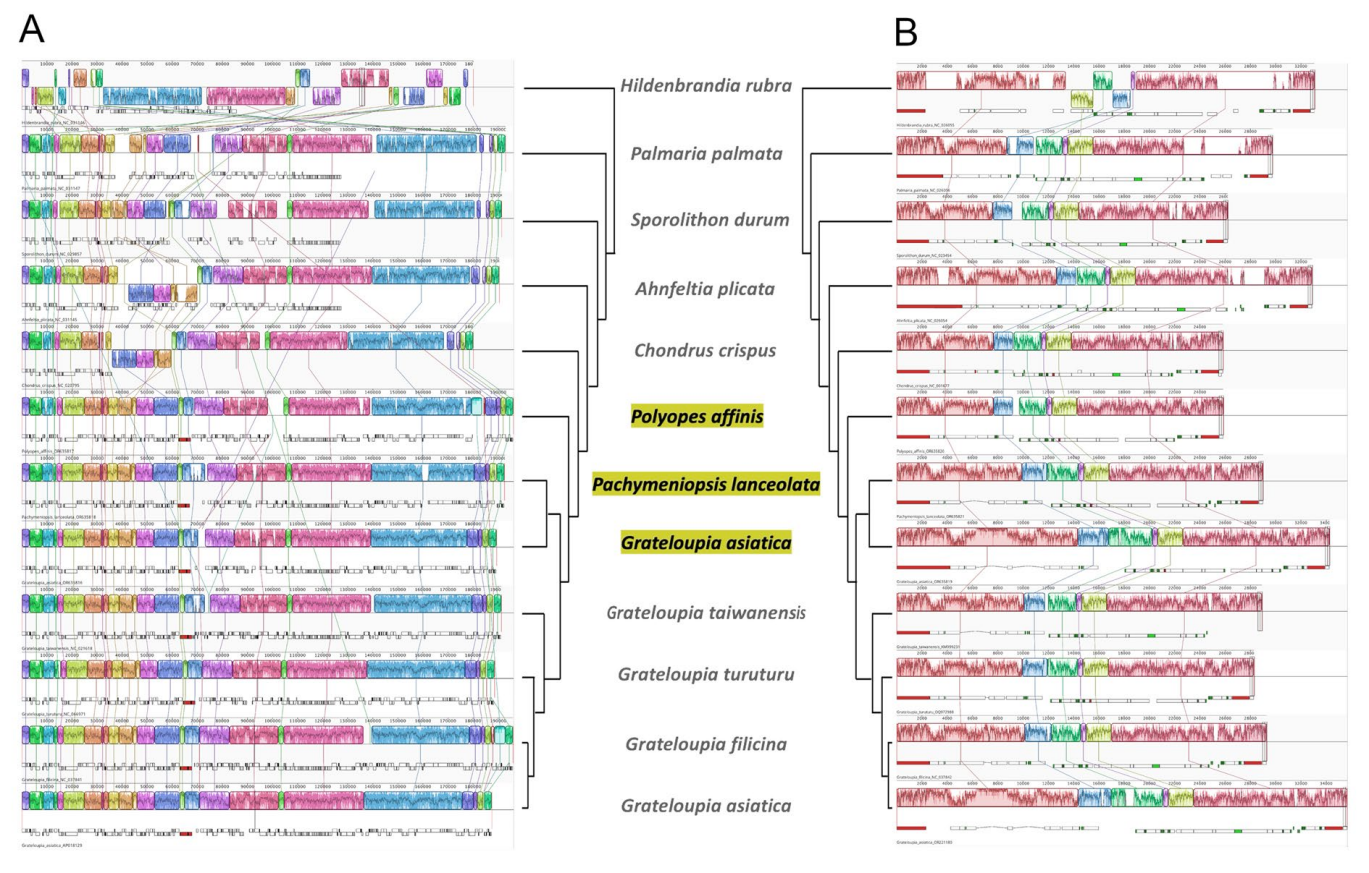
Comparison of the complete chloroplast genomes **A** and mitochondria genomes **B** of 12 species of Florideophyceae using the Mauve algorithm. Local co-linear blocks indicate syntenic regions

The synteny of genes was identified among species in class Florideophyceae. In class Florideophyceae, the order Halymeniales showed a highly conserved gene order among the species, and *Palmaria palmata* (order Palmariales) and *Sporolithon durum* (Sporolithales) had almost identical gene orders (Fig. 3A). Compared to other species in Florideophyceae, except for *Hildenbrandia rubra* (Hildenbrandiales), *Ahnfeltia plicata* (Ahnfeltiales) and *Chondrus crispus* (Gigartinales) had one inversion (from *rps6* to *ycf46*, a total of 31 genes) in their cp genomes. The mt genomes of species in Florideophyceae also showed highly conserved gene order except for *Hildenbrandia rubra* (Fig. 3B).

**Fig. 4:**
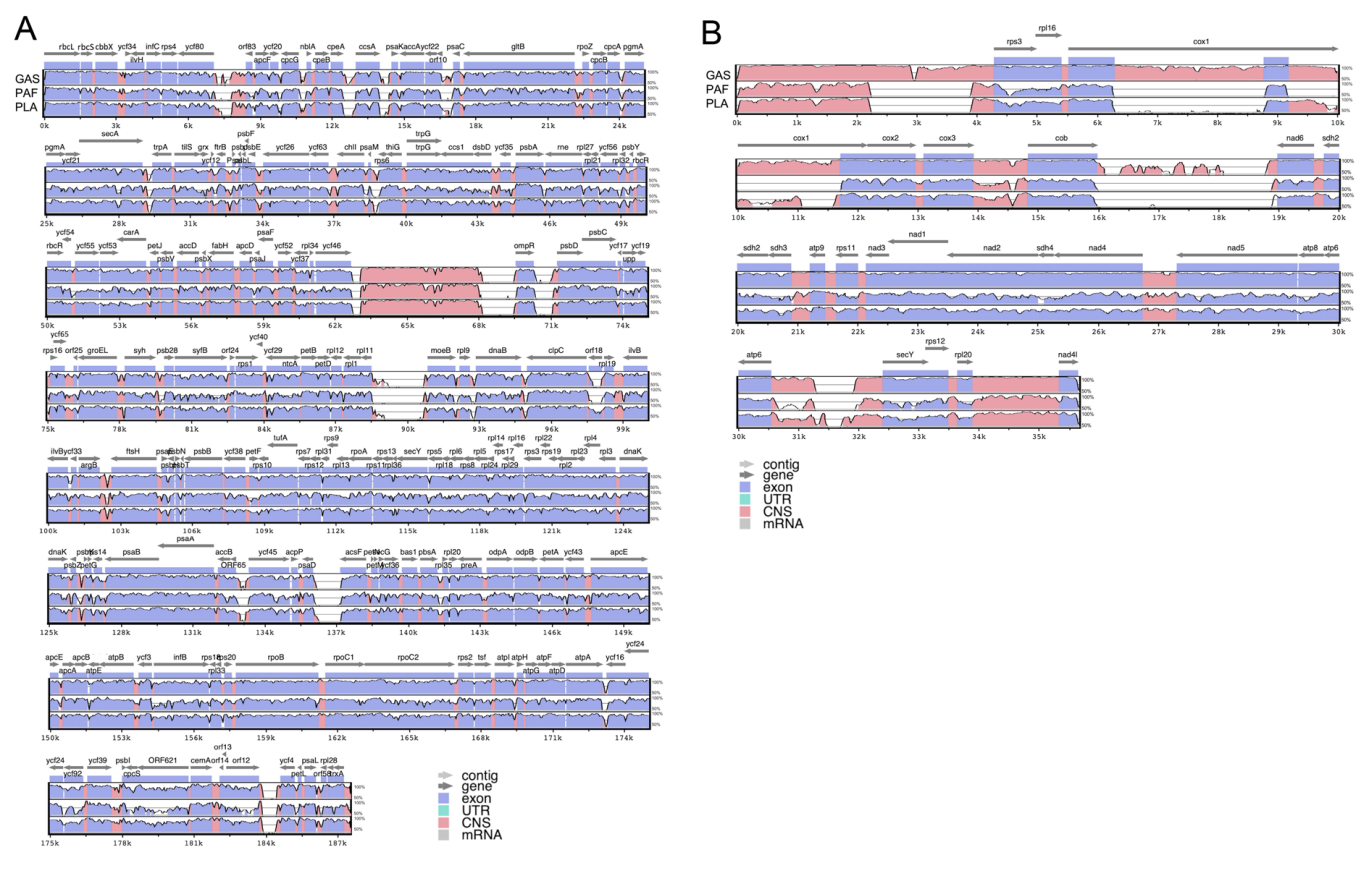
Sequence alignment and comparison of three species using mVISTA in chloroplast genomes **A** and mitochondrial genomes **B**. Previously reported organelle genomes of *G. asiatica* (AP018129.2 for cp genome and OR221180.1 for mt genome) were used as alignment references. *GAS* *G. asiatica*, *PAF* *P. affinis*, *PLA* *P. lanceolata*

We used mVISTA to identify organelle genomic divergence among the three species, with reference sequences of previously reported *G. asiatica* (Fig. 4). These results revealed that the gene-coding regions (purple) were more conserved compared to the non-coding regions (pink, except for rRNA regions around 63–68 k in cp genomes and 0–2.3 k, 22 k, and 34–35.3 k in mt genomes) in both cp and mt genomes. In particular, the mitochondrial *cox1* gene has two introns in *G. asiatica*, one intron in *P. lanceolata*, and no introns in *P. affinis*, as shown in Fig. 4B.

**Fig. 5:**
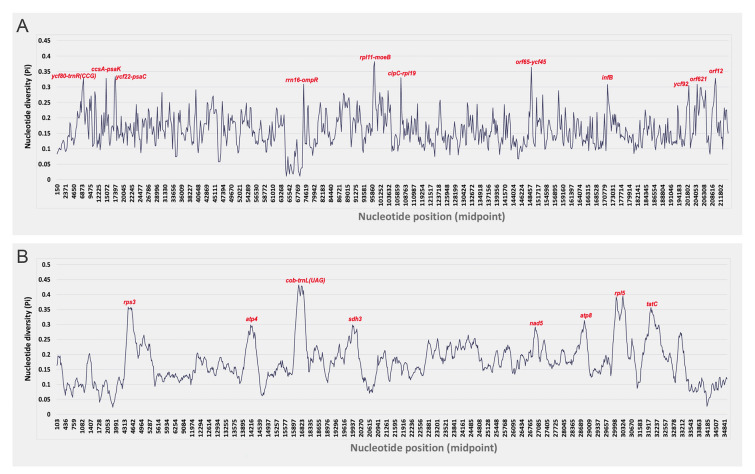
Sliding window analysis of the complete chloroplast genomes **A** and mitochondria genomes **B** of the three species

To further analyze the divergence hotspots of the organelle genomes among three species, a sliding window analysis of nucleotide diversity π (Pi) was performed. The Pi value of the three cp genomes ranged from 0.011 to 0.382 with the most divergent (Pi > 0.3) non-coding regions of *ycf80*-*trnR(CCG)*, *ccsA*-*psaK*, *ycf22*-*psaC*, *rrn16*-*ompR*, *rpl11*-*moeB*, *clpC*-*rpl19* and *orf65*-*ycf45* and the protein-coding regions of *infB*, *ycf92*, *orf621*, and *orf12* (Fig. 5A). We also observed eight hotspots with high sequence variability (Pi > 0.29), including one non-coding region, *cob*-*trnL(UAG)*, and seven protein-coding regions of *rps3*, *atp4*, *sdh3*, *nad5*, *atp8*, *rpl5*, and *tatC* in the mt genomes (Fig. 5B). The Pi value of the three mt genomes ranged from 0.023 to 0.432.

**Fig. 6:**
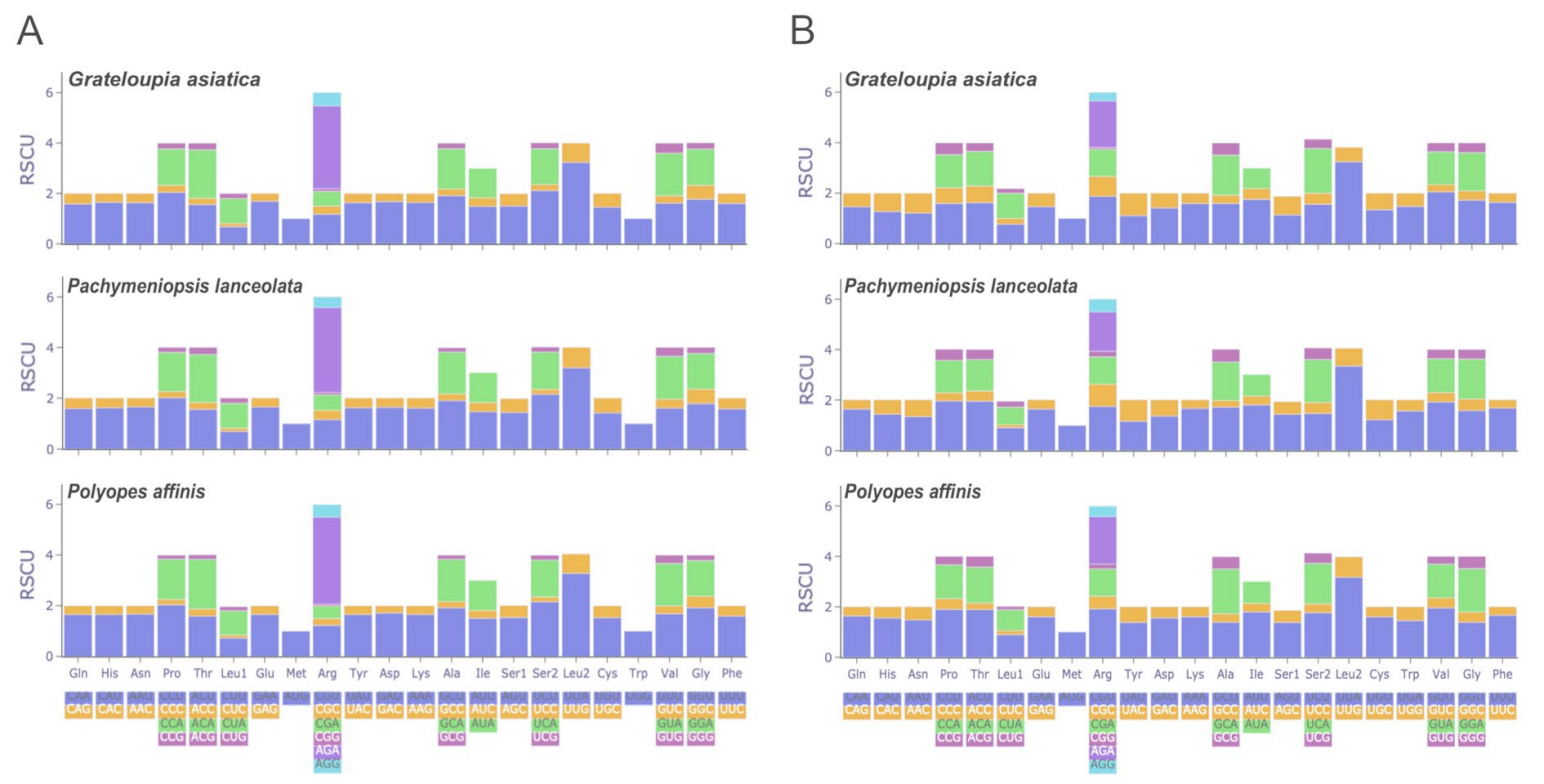
The relative synonymous codon usage (RSCU) in all protein-coding genes of chloroplast genomes **A** and mitochondrial genomes **B**

We estimated the RSCU for all PCGs in the organelle genomes of the three species (Supplementary Table S4) and plotted the results in Fig. 6. In the cp genomes, 204 PCGs consisted of 48,641 codons (*G. asiatica*), 202 PCGs consisted of 48,642 codons (*P. lanceolata*), and 199 PCGs consisted of 47,893 codons (*P. affinis*). Leucine was the predominant amino acid in PCGs with a count of 5067 (10.42% in *G. asiatica*), 5037 (10.36% in *P. lanceolata*), and 4995 (10.43% in *P. affinis*), followed by isoleucine (average counts of 4914, 10.16%), lysine (average counts of 3507, 7.25%), and serine (average counts of 3424, 7.08%), while cysteine (average counts of 486, 1%) was the least used amino acid among the cp genomes of the three species. The two most frequently used codons were AAA (lysine) and UUA (leucine). AAU (asparagine) and AUU (isoleucine) were the third and fourth most frequently used codons in *P. lanceolata* and *P. affinis*, whereas they were the fourth and third most frequently used codons in *G. asiatica*. The codons AGA (arginine) and UUA were found to have the highest RSCU values (average values of 3.35 and 3.24, respectively). The mt genomes of all three species contained 25 PCGs, with 6088 codons (*G. asiatica*), 6079 codons (*P. lanceolata*), and 6066 codons (*P. affinis*). Like the cp genomes, the mt genomes also contained leucine as the most abundant amino acid, followed by isoleucine (average counts of 918 and 601, respectively). Serine was the third most abundant amino acid in the cp genomes but fourth in the mt genomes, with phenylalanine being the third most abundant amino acid (average counts of 548 and 505 for phenylalanine and serine, respectively). UUA was identified as the most frequently used codon and had the highest RSCU value in mt genomes (average counts of 497 and RSCU value of 3.25). In all cases, it was confirmed that A/U bases appeared preferentially at the third position of the codon, rather than C/G, and all codons with RSCU > 1 ended in A or U.

The non-synonymous (Ka) and synonymous (Ks) substitutions and their ratios (Ka/Ks) were calculated for the organelle genomes of seven Halymeniaceae accessions to evaluate natural selection pressure on nucleotides. The three chloroplast genes in the four comparisons (*psbV* in *G.asiatica* [AP018129.2] vs. *G. filicina*, *ycf33* in *G. turuturu* vs. *P. lanceolata*, *orf621* in *G. filicina* vs. *G. turuturu*, and *G. asiatica* [AP018129.2] vs. *G. turuturu*) had Ka/Ks values over 1.0, but were not statistically significant (Fisher’s test *P*-value > 0.05), which were consequently excluded from the results. The significant pairwise Ka/Ks values between species are plotted as a heatmap in Supplementary Fig. S1. Two genes (cp gene *ycf92* in *G. turuturu* vs. *P. affinis*, and *G. taiwanensis* vs. *G. turuturu*, and mt gene *rpl20* in *G. filicina* vs. *P. affinis*) had Ka/Ks values over 0.5, and in all other cases, the Ka/Ks values were less than 0.5.

### Phylogenetic analysis

Phylogenetic relationships were inferred from the aligned, trimmed, and concatenated sequences of 194 and 23 PCGs of cp and mt genomes, respectively (Supplementary Table S2). The cp PCGs were obtained from 26 NCBI GenBank accessions and mt PCGs were obtained from 30 accessions. The phylogenetic trees were reconstructed using the concatenated cp PCGs sequence matrix (Supplementary Fig. S2a), mt PCGs sequence matrix (Supplementary Fig. S2b), and combined matrix of the cp and the mt matrices (Supplementary Fig. S2c) with the best-fit models of GTR + F + I + R5, TIM3 + F + I + R7, and GTR + F + R7, respectively. To construct a combined phylogenetic tree, the combined sequence matrix was concatenated by selecting species for which both cp and mt accessions were available (25 species). According to the results of the cp-based phylogenetic tree, species of the order Halymeniales (including four genera: Grateloupia, Halymenia, Pachymeniopsis, and Polyopes) were divided into two clades (*P. affinis* and *H. maculata* were clustered together and separated from other Halymeniales species), but were closely clustered. In the mt-based ML tree, the Halymeniales species were also divided into two clades, with exception of one species (previously reported *P. affinis*, NC_63816). The other two Polyopes species were grouped into the same clade as Halymeniales species, but NC_63816 was grouped into a different clade together with *Chondrus crispus* (Gigartinales). Together with the combined tree results, Polyopes species seems to be evolutionarily distant among Halymeniales species. In all three ML trees, all species belonging to the class Florideophyceae clustered with the order Bangiales (one of the orders of the class Bangiophyceae).

### Development of species-specific markers for the identification of three Halymeniaceae species

**Fig. 7:**
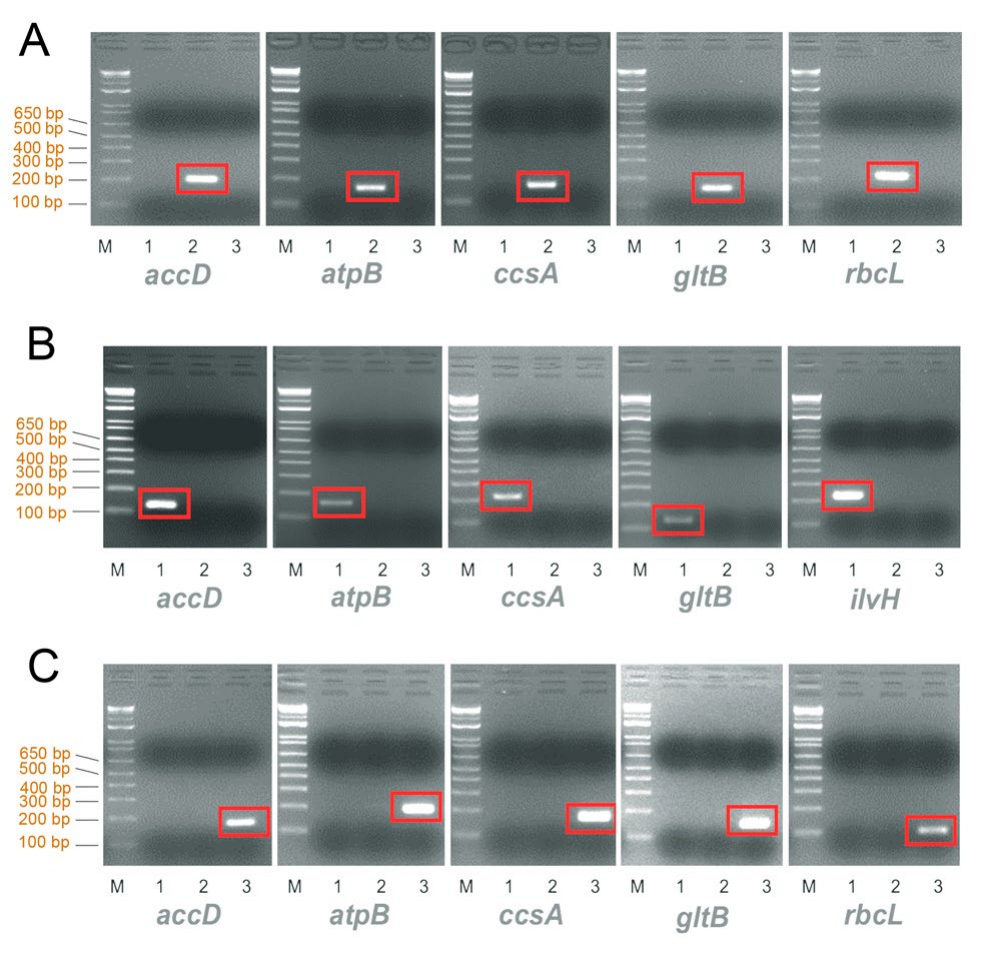
Development of quantitative PCR based on the single nucleotide polymorphisms (SNPs) of chloroplast PCGs. Target species are **A**: *G. asiatica*, **B**: *P. lanceolata*, and **C**: *P. affinis*. Lane 1: *P. lanceolata*, 2: *G. asiatica*, 3: *P. affinis*, M: DNA ladder

The PCG sequences of the three cp genomes were aligned to detect the SNPs, and we developed 15 species-specific markers (five markers per species) from six genes (*accD*, *atpB*, *ccsA*, and *gltB* for all the three species, *rbcL* for *G. asiatica* and *P. affinis*, and *ilvH* for *P. lanceolata*) (Fig. 7). The markers were designed with PCR product sizes ranging from 103 to 223 bp, cut-off Ct ranging from 24 to 26 cycles, and melting temperatures ranging from 56 to 61.4 ℃ (Supplementary Table S5). We focused on cp genomes for the development of SNP-based markers because cp genomes have more conserved sequences than those of mt genomes.

## Discussion

### Overview of organelle genome assembly

Organelle genomes play an important role in DNA barcoding, molecular marker development and phylogenomics (Cai et al. 2021; Li et al. 2021; Yang and Kim 2015). In this study, we obtained the complete organelle genomes of *Grateloupia asiatica*, *Pachymeniopsis lanceolata* and *Polyopes affinis*, which included the first cp genome of *P. lanceolata* and *P. affinis* and the first mt genome of *P. lanceolata*. Our results provided insights into the genomic architecture, gene content and structural variations, forming a basis for further studies.

### Gene composition, pseudogenization and selection pressure

Gene composition across the three species was highly conserved, particularly in the mitochondrial genomes. The missing PCGs were not found in the mitochondria, but appeared in the cp genomes, and most of them were functionally unknown genes, such as *orf13*/*16*/*18*/*24*/*25*/*48*/ and *ycf21*/*56*. *P. affinis* exhibited pseudogenization of *grx*, leading to truncated functionality. This aligns with the previous finding, pseudogenization commonly occurs in cp genomes (Frailey et al. 2018; Liu et al. 2020; Scobeyeva et al. 2021). These findings shed light on the evolutionary dynamics of the chloroplast genome in Florideophyceae and highlighted the potential genetic marker development for further studies.

Ka/Ks analysis supported that all PCGs are experiencing purifying selection, with no gene showing a significant Ka/Ks > 1. Most genes had Ka/Ks values < 0.1, suggesting the strong evolutionary constraints for maintaining functionality. These findings aligned with those of previous studies emphasizing the evolutionary stability of organelle genomes in Florideophyceae (Wang et al. 2010; Wu et al. 2020; Yu et al. 2019; Zhou et al. 2023).

Codon usage bias analysis revealed a preference for codons ending in A or U, which is consistent with the trend observed among red algae (Lee et al. 2022; Lu et al. 2023; Munyao et al. 2020; Xu et al. 2023). This bias reflects the potential optimization of translational efficiency and adds to our understanding of the evolutionary dynamics influencing organelle genomes (Plotkin and Kudla 2011; Sharp and Matassi 1994; Yang et al. 2023; Yao et al. 2023; Zhou et al. 2016).

### Structural variation in organelle genomes

Unexpectedly, chloroplast genomes in Florideophyceae displayed more structural rearrangements than mitochondrial genomes (Fig. 3), in contrast to the typical trends observed in other taxa (Smith and Keeling 2015). In particular, an inverse region of 31 genes, *rps6* to *ycf46*, was identified in *Ahnfeltia plicata* and *Chondrus crispus*. Although there are still many uncertainties about the gene rearrangements, repeat elements are known to be one of the important factors in such rearrangements (Maréchal and Brisson 2010; Ogihara et al. 1988; Tremblay-Belzile et al. 2015; Zampini et al. 2015; Zhang et al. 2012). In the cp genomes of *Palmaria palmata* and *Ahnfeltia plicata*, inverted rRNA gene (rRNA operon) repeats were identified on both sides of the region from *rps6* to *ycf46*. Lee et al. (2016) illustrated the structures of the cp genomes of red algae and seed plants, and divided the cp genomes of Florideophyceae into three types according to their structural characteristics. Consistent with their results, *Palmaria palmata*, *Sporolithon durum*, and seven other species belonging to Halymeniales, including the three species assembled in this study, were the R2-type, and two species with an inversion region (*A. plicata* and *C. crispus*) were the R1-type. The difference between the R1- and R2-type is that the inversion region is identical to the region between the inverted repeat rRNA operons. In both R1- and R2-types of cp genomes, there are species in which one set of rRNA operons was lost during the evolutionary process (such as *S. durum* and Halymeniale in R2-type, and *C. crispus* in R1-type). Many cp genomes found in diaphoretickes (Archaeplastida, by primary endosymbiosis, such as Viridiplantae, and the Sar supergroup, by secondary endosymbiosis, such as brown algae) have a typical quadripartite architecture consisting of a large single copy (LSC), a small single copy (SSC), and inverted repeats (IRs), where the IRs most likely contain an rRNA operon and several genes or pseudogenized sequences. Loss of IRs occasionally occurs in cp genomes; however, information regarding its cause is limited (Bedoya et al. 2019; Jin et al. 2020a, b).

### Sequence similarity and evolutionary hotspots

Sequence similarity analysis demonstrated lower identity in mt genomes (73.4%) than in cp genomes (81.1%) across the seven Halymeniales species. The three species used in this study showed average similarities of 74% and 66.54% in the cp and mt genomes, respectively, and the average similarity of all genes (including PCGs, rRNAs, and tRNAs) was 84.8% and 70.1% in the cp and mt genomes. mVISTA and sliding window analyses identified divergent hotspots (Figs. 4 and 5), highlighting regions with high variability that could be valuable for marker development. The lower similarity in mt genomes may reflect a faster evolutionary rate, providing a fine-scale resolution for species-level differentiation (Smith and Keeling 2015).

### Phylogenetic insights

The phylogenetic analyses revealed the relationship among the species in Florideophyceae and highlighted the close association of *P. lanceolata* with Grateloupia species. The newly sequenced cp genome of *G. asiatica* (OR635816) was found to be distant from the previously reported cp genome of *G. asiatica* (AP018129), possibly because AP018129 is a “nearly” complete genome. The combined cp and mt genome-based trees closely resembled the cp-based tree, likely due to the higher number of PCGs in chloroplast genomes. These findings highlight the utility of chloroplast genomes in resolving evolutionary relationships within Florideophyceae.

Six species belonging to order Bangiales and class Bangiophyceae (blue boxes in Supplementary Fig. S2) were clustered together with the species of the class Florideophyceae (red boxes in Supplementary Fig. S2), used to reconstruct phylogenetic trees based on both cp and mt genomes. The taxonomic classifications of the species used in this study were according to NCBI taxonomy, but previous studies differed from NCBI’s classification (Lee et al. 2016; Park et al. 2023; Yang et al. 2015). In the results of these researchers, six other species (*C. chilensis*, *C. caldarium*, *D. grisea*, *E. coxiae*, *G. sulphuraria*, and *P. aerugineum*) belonging to the order Bangiophyceae (excluding Bangiales) were classified into different orders such as Cyanidiophyceae or Porphyridophyceae. Such inconsistencies reinforce the need for an integrative approach that combines genomic data with taxonomic frameworks to resolve ambiguities. The inclusion of newly sequenced genomes in this study provides a foundation for future research on the evolutionary divergence and adaptation mechanisms in red algae.

### Species-specific marker development

The high AT content and repetitive nature of cp genomes present challenges for marker development. To overcome this limitation, we targeted protein-coding genes (PCGs) in chloroplast genomes to design species-specific PCR markers. These efforts resulted in 15 specific markers, five in each species, that could distinguish the studied species. These markers are important to ensure species identification in food and medicinal applications (Hardouin et al. 2022; Shabaka and Moawad 2021; Wang et al. 2020), particularly for red algal resources that are prone to misuse or misidentification during processing. The successful development of these markers highlights the practical application of genomic data to address the challenges associated with species identification. They also provided a scalable framework for marker development in other red algal species.

### Future perspectives

Our findings in this study highlighted the importance of organelle genomics in advancing our understanding of Florideophyceae diversity and resolving their evolutionary relationship. The newly sequenced organelle genomes provided foundational data for filling in the gaps in red algal research. Expanding organelle genomic data across underrepresented taxa could refine our understanding of red algal evolution and facilitate further phylogenetic studies.

## Supplementary Information

Below is the link to the electronic supplementary material.

Supplementary file1 (RAR 434 KB)

Supplementary file2 (DOCX 225 KB)

Supplementary file3 (DOCX 455 KB)

## Data Availability

The NCBI GenBank accession numbers of organellar genomes of the three species are shown below: 1. *Grateloupia asiatica* chloroplast genome: OR635816.1 (and registered in NCBI Reference Sequence: NC_084248.1). 2. *Grateloupia asiatica* mitochondrial genome: OR635819.1. 3. *Pachymeniopsis lanceolata* chloroplast genome: OR635818.1 (and registered in NCBI Reference Sequence: NC_084250.1). 4. *Pachymeniopsis lanceolata* mitochondrial genome: OR635821.1 (and registered in NCBI Reference Sequence: NC_084251.1). 5. *Polyopes affinis* chloroplast genome: OR635817.1 (and registered in NCBI Reference Sequence: NC_084249.1). 6. *Polyopes affinis* mitochondrial genome: OR635820.1. 7. Raw genome sequencing reads of *Grateloupia asiatica*: SRR26313966 in SRA. 8. Raw genome sequencing reads of *Pachymeniopsis lanceolata*: SRR26313964 in SRA. 9. Raw genome sequencing reads of *Polyopes affinis*: SRR26313965 in SRA.
