# Supplementary material for: Species-specific marker development for accurate identification of three red algae (Grateloupia asiatica, Pachymeniopsis lanceolata and Polyopes affinis) based on complete organelle genomes
Source: Mar Life Sci Technol. 2025 Oct 28;7(4):717–29. doi: 10.1007/s42995-025-00327-4 (PMC12662926; doi:10.1007/s42995-025-00327-4)
Supplement: Supplementary file 2 — Supplementary file2 (DOCX 225 KB) [file 42995_2025_327_MOESM2_ESM.docx]

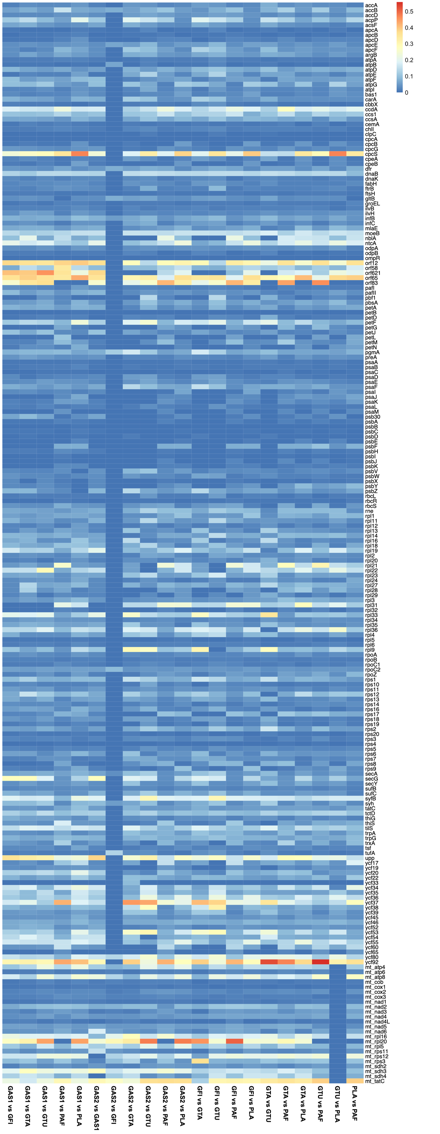


Fig. S1. Heatmap of Ka/Ks ratios between every compared species in 194 chloroplast genes and 24 mitochondria genes. The name of mitochondria genes were modified to start with ‘mt_’.

GAS1: *G. asiatica* (this study), GAS2: *G. asiatica* (previously reported), GFI: *G. filicina*, GTA: *G. taiwanensis*, GTU: *G. turuturu*, PAF: *P. affinis*, PLA: *P. lanceolata*.
