# Supplementary material for: Species-specific marker development for accurate identification of three red algae (Grateloupia asiatica, Pachymeniopsis lanceolata and Polyopes affinis) based on complete organelle genomes
Source: Mar Life Sci Technol. 2025 Oct 28;7(4):717–29. doi: 10.1007/s42995-025-00327-4 (PMC12662926; doi:10.1007/s42995-025-00327-4)
Supplement: Supplementary file 3 — Supplementary file3 (DOCX 455 KB) [file 42995_2025_327_MOESM3_ESM.docx]

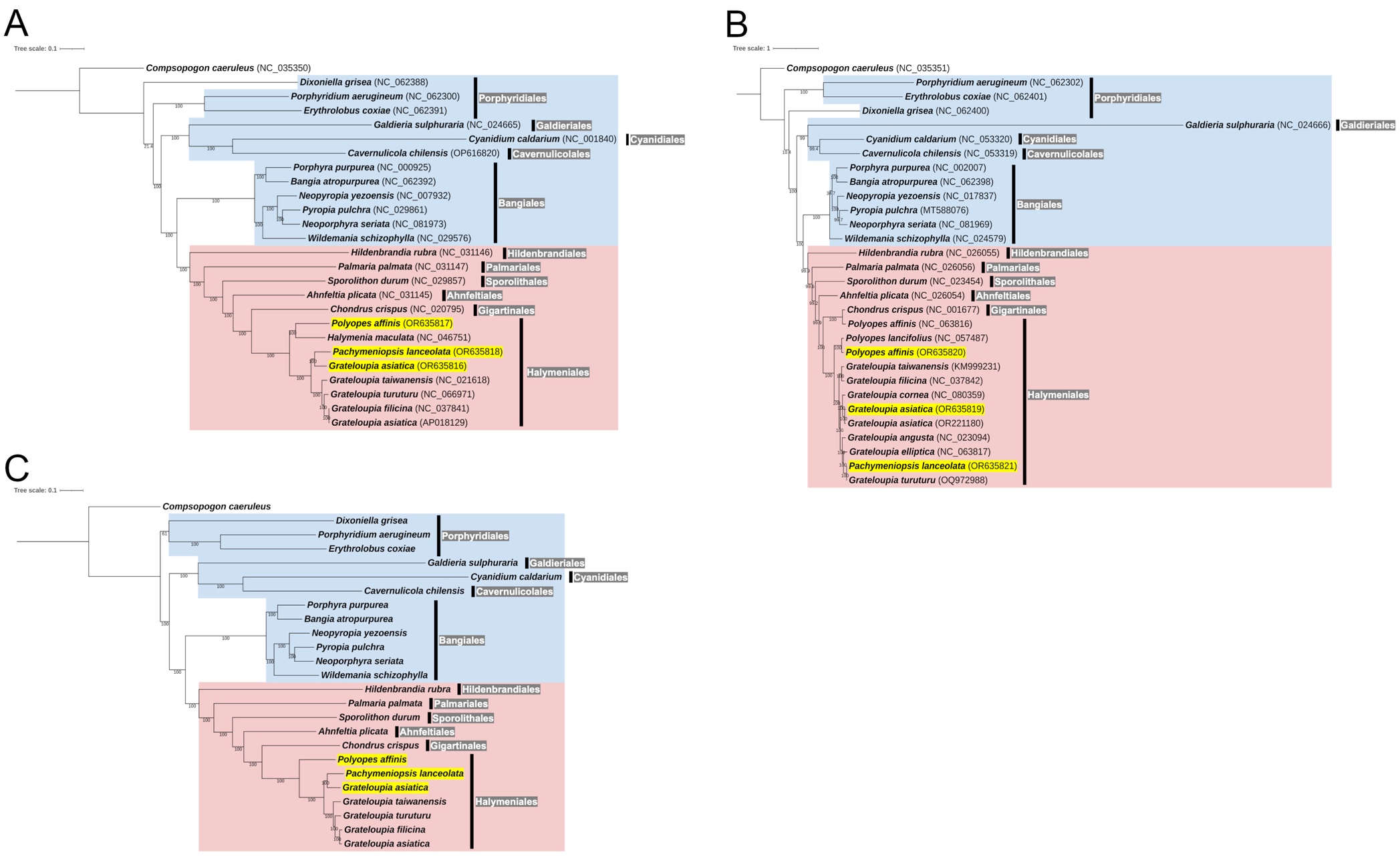


Fig. S2. The phylogenetic tree constructed using 194 protein-coding genes of the chloroplast genome for 25 accessions and one outgroup (A), from 23 protein-coding genes of mitochondrial genomes for 17 accessions and one outgroup (B), and from the combined 194 chloroplast protein coding genes and 23 mitochondrial protein-coding genes for 24 accessions, for which both chloroplast and mitochondria genomes were available (C). Species belonging to the class Bangiophyceae are indicated in a blue box and species belonging to the class Florideophyceae are indicated in a red box.
